# Towards Resolving the Pro- and Anti-Tumor Effects of the Aryl Hydrocarbon Receptor

**DOI:** 10.3390/ijms19051388

**Published:** 2018-05-07

**Authors:** Supraja Narasimhan, Elizabeth Stanford Zulick, Olga Novikov, Ashley J. Parks, Jennifer J. Schlezinger, Zhongyan Wang, Fabrice Laroche, Hui Feng, Francesca Mulas, Stefano Monti, David H. Sherr

**Affiliations:** 1 Department of Pathology and Laboratory Medicine, Boston University School of Medicine, 72 East Concord St., Boston, MA 02118, USA; supraja.narasimhan@gmail.com; 2 Biological Sciences and Biotechnology Programs, Northeastern University, Boston, MA 02115, USA; e.zulick@northeastern.edu; 3 Molecular and Translational Medicine Program, Department of Medicine, Boston University School of Medicine, 72 East Concord St., Boston, MA 02118, USA; olga@novatok.com; 4 Sage Therapeutics, 215 1rst St., Cambridge, MA 02142, USA; ashley.j.parks@gmail.com; 5 Department of Environmental Health, Boston University School of Public Health, 72 East Concord St., Boston, MA 02118, USA; jschlezi@bu.edu (J.J.S.); wangzhy@bu.edu (Z.W.); 6 Departments of Pharmacology and Medicine, Cancer Center, Boston University School of Medicine, 72 East Concord St., Boston, MA 02118, USA; fabrice75005@gmail.com16 (F.L.); huifeng@bu.edu (H.F.); 7 Department of Pediatrics, University of California, San Diego, CA 92093, USA; fra.mulas@gmail.com; 8 Division of Computational Biomedicine, Department of Medicine, Boston University School of Medicine, 72 East Concord St., Boston, MA 02118, USA; smonti@bu.edu

**Keywords:** aryl hydrocarbon receptor, cancer, AHR agonist, AHR antagonist, cancer therapeutics

## Abstract

We have postulated that the aryl hydrocarbon receptor (AHR) drives the later, more lethal stages of some cancers when chronically activated by endogenous ligands. However, other studies have suggested that, under some circumstances, the AHR can oppose tumor aggression. Resolving this apparent contradiction is critical to the design of AHR-targeted cancer therapeutics. Molecular (siRNA, shRNA, AHR repressor, CRISPR-Cas9) and pharmacological (AHR inhibitors) approaches were used to confirm the hypothesis that AHR inhibition reduces human cancer cell invasion (irregular colony growth in 3D Matrigel cultures and Boyden chambers), migration (scratch wound assay) and metastasis (human cancer cell xenografts in zebrafish). Furthermore, these assays were used for a head-to-head comparison between AHR antagonists and agonists. AHR inhibition or knockdown/knockout consistently reduced human ER^−^/PR^−^/Her2^−^ and inflammatory breast cancer cell invasion, migration, and metastasis. This was associated with a decrease in invasion-associated genes (e.g., *Fibronectin*, *VCAM1*, Thrombospondin, *MMP1*) and an increase in *CDH1/E-cadherin*, previously associated with decreased tumor aggression. Paradoxically, AHR agonists (2,3,7,8-tetrachlorodibenzo-*p*-dioxin and/or 3,3′-diindolylmethane) similarly inhibited irregular colony formation in Matrigel and blocked metastasis in vivo but accelerated migration. These data demonstrate the complexity of modulating AHR activity in cancer while suggesting that AHR inhibitors, and, under some circumstances, AHR agonists, may be useful as cancer therapeutics.

## 1. Introduction

The earliest studies on the aryl hydrocarbon receptor (AHR) focused on its role as an environmental chemical sensor and mediator of the toxic effects of polycyclic aromatic hydrocarbons, dioxins, and planar polychlorinated biphenyls, many of which are classified as human carcinogens [1]. However, the demonstration that the AHR is highly conserved throughout evolution [2] suggested that the AHR plays an important role in some “normal” biological activities. In a corollary to these studies, an accumulating body of evidence indicates that the AHR is important in the progression of a variety of cancer types [3,4,5,6,7,8,9,10,11,12,13,14,15,16]. For example, the AHR is hyper-expressed and “constitutively” active (likely a reflection of the presence of endogenous AHR ligands [13,17,18]) in a diverse array of human cancers including glioblastomas [18], adult T cell leukemias, and oral cavity, lung, liver, prostate and Her2^+^ breast adenocarcinomas [6,17,18,19,20,21,22,23,24,25]. However, because AHR activity is highly context (tissue type, cell type, ligand)-specific [10,26], predicting how the “Janus-faced” AHR [27] will act in any given system can be problematic. For example, it is sometimes unclear if the AHR promotes or inhibits tumor aggression in any given tumor type. In support of the AHR driving tumor aggression, AHR knockdown in lung adenocarcinoma [28] or oral squamous cell carcinoma [17], or depletion of putative endogenous AHR ligands in glioblastoma [18] decreases anchorage-independent growth and/or invasion. Ectopic AHR expression increases tumor cell motility and invasion [25]. Knockdown of the AHR repressor (AHRR), a putative tumor suppressor, increases anchorage-independent growth and tumor formation in lung cancer xenografts [29]. In contrast, the AHR has been reported to exhibit anti-tumorigenic effects in prostate [30], stomach [31] liver [9] pancreatic [32], and breast [33] cancers. A direct comparison between outcomes in these system has been confounded by the use of a variety of readouts (e.g., tumor growth or migration assays), AHR modulators (inhibitors, agonists, molecular knockdowns) and tumor types.

Given the increasingly frequent suggestion that AHR modulators (inhibitors or agonists) represent a new class of targeted cancer therapeutics [6,34,35,36,37,38,39,40], it is critical that additional studies be performed to determine, with a given set of readouts, tumor types and experimental models, if both AHR inhibition and hyper-activation generate similar or, paradoxically, opposite outcomes. Here, we address this issue by employing a set of molecular and pharmacological approaches to AHR modulation in invasion, migration, and metastasis assays to attempt to confirm the anti-tumorigenic effects of AHR inhibitors and to compare AHR agonists and antagonists head-to-head.

## 2. Results

### 2.1. Inhibition of AHR Expression or Activity Significantly Reduces Irregular Colony Formation by Human Mammary Tumor Cells in 3D Cultures

Rat and mouse primary mammary tumors and human mammary tumor cell lines are characterized by hyperexpression of constitutively active AHR [7,41,42,43,44,45,46]. Previously, we postulated that constitutive AHR activity, driven in part by the production of μM quantities of the endogenous tryptophan-derived ligands kynurenine and xanthurenic acid [17], is causally linked to breast cancer invasion [12,21]. From this it would be predicted that AHR inhibition would reduce multiple measures of tumor aggression. However, some studies suggest that AHR inhibition might generate the opposite outcome, calling into question the use of AHR inhibitors for cancer treatment [9,30,31,32,33,37].

**Figure 1:**
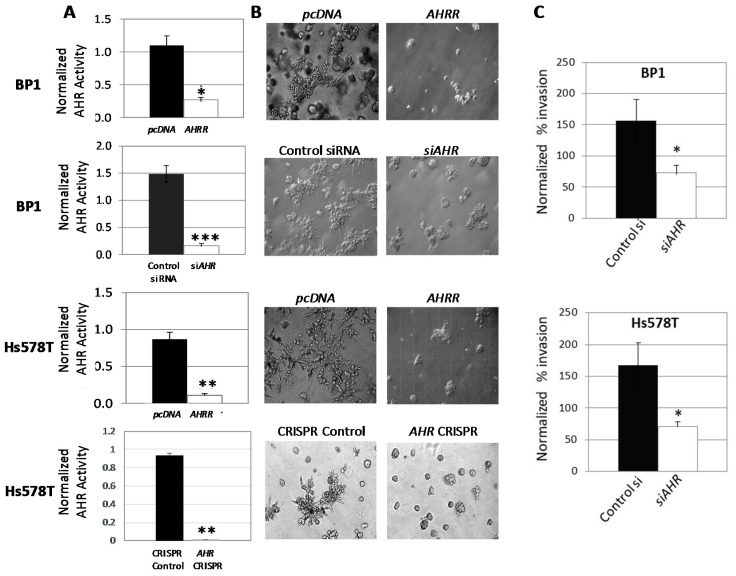
AHR downregulation reduces irregular colony formation in 3D Matrigel cultures and inhibits invasion in Boyden chambers. (**A**) Wild-type BP1 and Hs578T cells were co-transfected with AHRE-driven firefly luciferase reporter (*pGudLuc*), *phRL-TK* (*renilla*), *and pcDNA* control vector, *AHRR* plasmid, control scrambled siRNA, or *AHR*-specific siRNA as indicated and incubated for 24–48 h. Hs578T cells transduced with a CRISPR-Cas9 control vector or with an *AHR*-specific CRISPR-Cas9 construct were similarly transfected with *pGudLuc* and *phRL-TK*. Cells were harvested and pGudLuc luciferase activity normalized to renilla luminescence. Data from 3–5 independent experiments are expressed as normalized mean luciferase activity + SE. * *p* < 0.02, ** *p* < 0.01, *** *p* < 0.001 relative to controls using the Student’s *t*-test. (**B**) BP1 and Hs578T cells were transfected with *pcDNA* control plasmid, *AHRR*, control scrambled *siRNA*, or *AHR*-specific *siRNA* plasmids and plated 24 h later in 3D Matrigel cultures in duplicate wells. Hs578T cells transduced with a CRISPR-Cas9 control vector or with an *AHR*-specific CRISPR-Cas9 construct were similarly cultured in Matrigel. Images were taken on days 4–7 of culture at 400× magnification and are representative of a minimum of two independent experiments with duplicates in each experiment. (**C**) BP1 (**top**) and Hs578T (**bottom**) cells were transfected with scrambled control *siRNA* or *AHR*-specific *siRNA* for 24 h before serum starvation for 18 h. Cells were harvested, counted, resuspended in serum-free media, and plated in triplicate in the upper chamber of Boyden chambers. Serum-containing, complete medium was placed in the lower chamber. Chambers were separated by 8 μM Matrigel-coated membranes. Invasive cells in the lower chamber of individual wells were dissociated from the membrane 48 h later, lysed and stained with CyQuant GR dye and fluorescence quantified. Data pooled from 4–5 independent experiments are presented as the mean percent invasion normalized to untransfected controls + SE, * *p* < 0.05 using the Student’s *t*-test as compared with the scrambled *siRNA*-transfected control group.

To confirm the role of the AHR in an aggressive cancer phenotype, AHR expression or activity was downregulated in human ER^−^/PR^−^/Her2^−^ (triple-negative breast cancer/TNBC) BP1 and Hs578T cell lines by transfection of an AHR repressor plasmid (*AHRR*), *AHR*-specific siRNA or by *AHR* deletion through CRISPR-Cas9-mediated *AHR* gene editing. Estrogen receptor-negative (ER^−^) cells were used since there remains an unmet medical need for targeted therapeutics for ER^-^ breast cancers and since interpretation of outcomes involving the AHR in ER^+^ cells is confounded by the well-established cross-talk between the AHR and ER signaling pathways [47,48,49,50,51,52]. All three approaches to suppressing AHR activity significantly reduced baseline AHR-dependent luciferase reporter (pGudLuc) activity (Figure 1A). (AHR knockout by CRISPR-Cas9 in Hs578T cells was further confirmed in Western blots and by demonstrating a decrease in endogenous levels of AHR-regulated *CYP1B1*, Figure S1). Furthermore, all three molecular approaches consistently inhibited the ability of BP1 and/or Hs578T cells to form irregular, branching colonies in Matrigel (Figure 1B) in an established 3D model of tumor cell invasion [53]. That this change in colony phenotype reflected an ability of cells to invade through the Matrigel was supported by a significant decrease in the ability of both BP1 and Hs578T cells to migrate across a Matrigel-coated membrane in Boyden chambers after AHR knockdown with siRNA (Figure 1C). Similar results were obtained with the ER^-^/PR^-^/Her2^−^ inflammatory breast cancer (IBC)-derived SUM149 cells. 

Furthermore, in contrast to previous studies in which AHR expression was downregulated with *AHR*-specific *siRNA* in ER^+^ breast cancer lines [33], no differences were seen in the proliferation rates or viability (>95% by trypan blue and/or propidium iodide exclusion assays) of cells transfected with *AHRR* or *AHR*-specific *siRNA* or in which *AHR* was deleted by CRISPR-cas9 knockdown (Figure S2). No differences were seen in the number of tumor cells recovered from the Matrigel, supporting the conclusion that AHR inhibition does not affect cell growth or death rates under these conditions.

**Figure 2:**
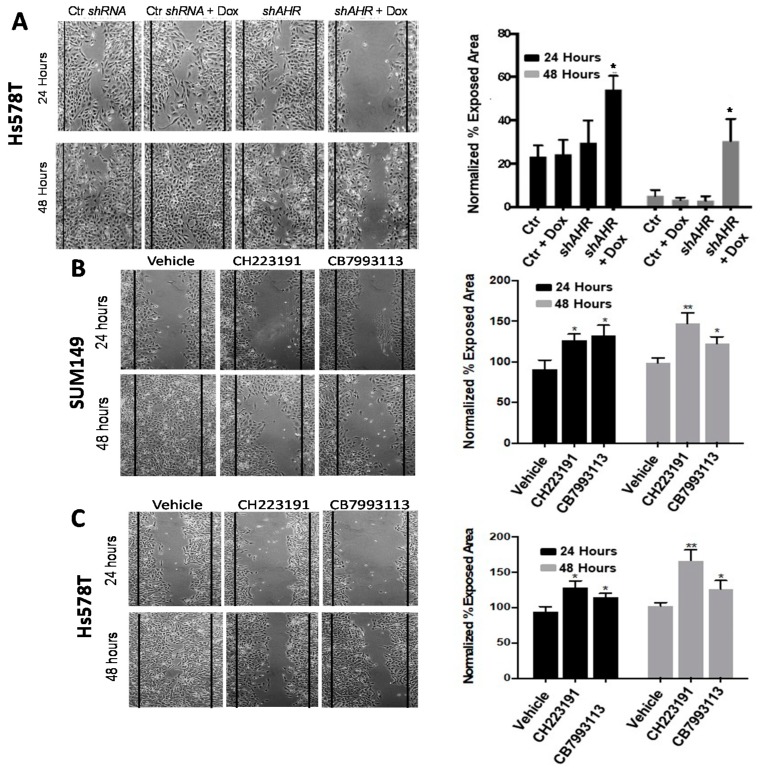
AHR inhibition with inducible shAHR or with AHR antagonists slows tumor cell migration. (**A**) Hs578T cells were transiently transfected with control scrambled *siRNA* or doxycycline-inducible *AHR*-specific *shRNA*, grown to confluence, and scratched with a pipette tip. Cultures were then treated with vehicle (PBS) or Dox as indicated, photographed at 100× magnification, and the percent exposed area digitally quantified at 24 and 48 h. Vertical black lines indicate the border of the scratch at time 0. Left: Representative images from a minimum of three independent experiments at 24 and 48 h are presented. Right: Data are quantified as the average percent exposed area + SE from a minimum of three independent experiments. * *p* < 0.05 compared with controls. (**B**,**C**) SUM149 (**B**) or Hs578T (**C**) cells were grown to confluence, scratched, and treated with vehicle (0.1% DMSO), 10 μM CH223191 or 10 μM CB7993113. Left: Representative images taken at 24 and 48 h from a minimum of three independent experiments. Right: Data are quantified as the percent exposed area + SE from a minimum of three independent experiments. * *p* < 0.05, ** *p* < 0.01 compared with vehicle controls.

To determine the effects of AHR knockdown on mammary tumor cell migration, Hs578T cells were transfected with a control scrambled *shRNA* or *AHR*-specific *shRNA* (*shAHR*). The transducing vectors also included a Doxycycline (Dox)-inducible TurboRFP coding sequence for inducing and tracking plasmid expression. High levels of TurboRFP, as assessed by flow cytometry and fluorescent microscopy, were seen within 24–48 h of Dox addition. *shAHR* induction significantly reduced nuclear and cytoplasmic AHR expression (Supplemental Figure S3A,B) and reporter activity (Supplemental Figure S3C). Control scrambled-*shRNA* or Dox-inducible *shAHR*-transfected cells were plated to confluence, scratched vertically across the wells, and cell migration rates quantified digitally in a 24–48 h “scratch-wound” assay in the presence or absence of Dox. While the control *shRNA* had no effect on migration in the presence or absence of Dox, and the *shAHR* had no effect on migration in the absence of Dox, Dox-induced *shAHR* significantly (*p* < 0.05) slowed Hs578T cell migration rate, as quantified by an increase in exposed area (Figure 2A). 

To extend these studies to a an IBC line and to pharmacological inhibitors of AHR activity, SUM149 and Hs578T cells were cultured in the scratch wound assay with vehicle (0.1% DMSO) or 10 μM of either of two competitive AHR inhibitors, CH223191 [54] or CB7993113 [55]. Both inhibitors significantly (*p* < 0.01, *p* < 0.05) reduced cell migration rates (increased exposed area at 24 and 48 h) (Figure 2B,C). As in the 3D Matrigel assays, these results were not due to changes in cell viability or proliferation as assayed by trypan blue or propidium iodide staining or ^3^H-thymidine incorporation. Similar results were obtained with the BP1 TNBC line.

These in vitro studies, using four molecular approaches (*AHRR*, *siRNA*, inducible *shRNA*, CRISPR/Ca9 gene editing) and two AHR-specific competitive inhibitors (CH223191, CB7993113), indicate that inhibition of AHR activity reduces two measures of tumor cell aggression, irregular colony growth characteristic of invasive cells and cell migration.

### 2.2. AHR Downregulation with Inducible shAHR Alters Expression of Multiple Tumor Invasion-Associated Genes

Tumor invasion is regulated by several genes that encode adhesion molecules controlling cell–cell or cell–extracellular matrix interactions and proteases capable of degrading the extracellular matrix [56,57,58,59]. Expression of some of these genes, particularly those encoding matrix metalloproteinases (MMPs), is influenced by AHR activity [60,61,62,63]. To further document that AHR inhibition initiates molecular changes consistent with a decrease in invasive potential, AHR knockdown studies were performed with Hs578T cells stably transduced with the Dox-inducible *shAHR* lentiviral vector used to generate data in Figure 2 and Figure S3. Cells were treated in 3D Matrigel cultures to more closely approximate interactions between malignant cells and the extracellular matrix that are known to influence tumor gene expression and cell function [64,65]. RNA extracted from *shAHR*-transduced Hs578T cells grown in Matrigel for 6 days in the absence or presence of Dox was evaluated by multiplex qPCR for expression of 84 invasion/migration-associated genes (Supplemental Table S1). AHR knockdown significantly altered levels of eight invasion-associated genes greater than 2-fold (*p* = 0.022–0.052) (Table 1). In particular, AHR knockdown resulted in a 5.07-fold increase in *CDH1* (E-cadherin) and a 3.65-fold decrease in *FN1* (fibronectin 1). An inverse relationship between E-cadherin and fibronectin 1 expression is characteristic of cells in transition between a non-invasive epithelial phenotype and an invasive mesenchymal stage where the former is characterized by relatively high E-cadherin and low fibronectin and the latter is characterized by relatively low E-cadherin and high fibronectin levels [66,67,68,69,70]. These results are reminiscent of our results with murine mammary tumor cell lines in which AHR expression correlated inversely with E-cadherin and positively with fibronectin levels [43,71]. AHR knockdown also resulted in a significant decrease in several genes associated with tumor invasion including those encoding VCAM1, thrombospondin, collagen type XIV, α1 and XV α1, and metalloproteinases 1 and 13. While AHR-regulation of metalloproteinase mRNA levels is suggestive of their role in AHR-driven metastasis, it should be noted that MMP control of invasion is often mediated by posttranscriptional processes that were not assayed here. Therefore, we cannot make firm conclusions about the lack of a role for the other MMPs in AHR-regulated invasion of cancer cell invasion.

**Figure 3:**
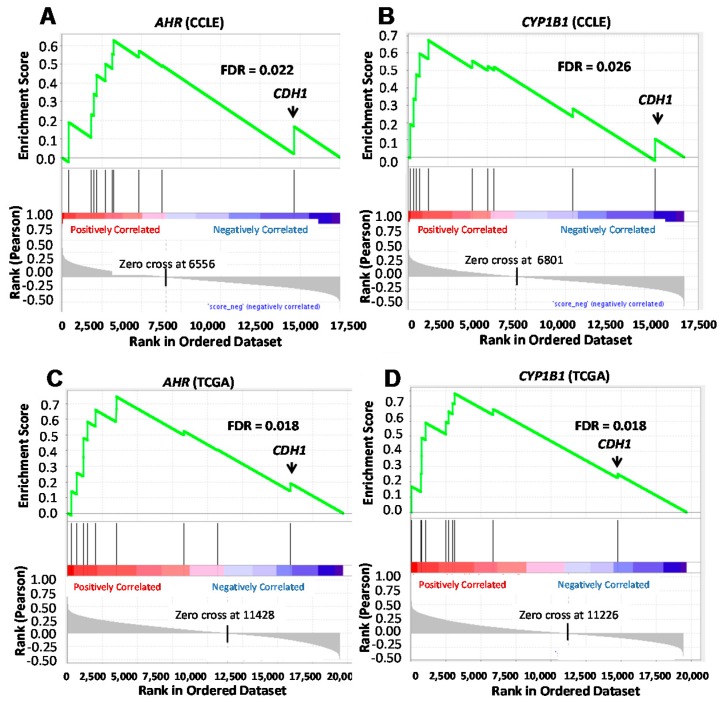
Gene set enrichment analysis of the correlation between *AHR* or *CYP1B1* and expression of a set of invasion-associated genes regulated in human breast cells after AHR knockdown using the CCLE and TCGA databases. The gene set enrichment analysis (GSEA) tool (http://www.broad.mit.edu/gsea) was used to correlate *AHR* (**A**,**C**) or *CYP1B1* (**B**,**D**) gene expression with expression of the gene set regulated following AHR knockdown in Hs578T cells (Table 1), as documented in the CCLE (**A**,**B**) or the TCGA (**C**,**D**) databases. Vertical black lines represent a given gene’s position in the ranked list incremented in the enrichment score statistic (“ES”, plotted in green). A significant positive correlation was demonstrated between *AHR* (FDR = 0.022) or *CYP1B1* (FDR = 0.026) and the invasion gene set in the CCLE database, with *CDH1* (E-cadherin) showing the lowest correlation value. Similarly, a significant positive correlation was demonstrated between *AHR* (FDR = 0.018) or *CYP1B1* (FDR = 0.018) and the invasion gene set in the TCGA database, with *CDH1* again showing the lowest correlation value.

Multiple factors determine the expression of these genes and primary human mammary tumors are, on a molecular level, very heterogeneous. However, if the changes in expression of the AHR-linked genes identified above in individual cell lines reflect the contribution of the AHR to primary human mammary tumor invasion in general, then one might expect to see a correlation between expression of these invasion-associated genes and AHR levels and/or AHR activity in other breast cancer cell lines and in primary human breast cancers. To test this hypothesis, we performed gene set enrichment analyses (GSEA) using transcriptomic data compiled by the Broad Institute on 60 human breast cancer cell lines in the *Cancer Cell Line Encyclopedia* (CCLE) [72]. Although it is recognized that several factors contribute to *CYP1B1* expression [73,74,75], *CYP1B1* levels were chosen as an approximation of AHR activity for three reasons: (1) We and others previously demonstrated that baseline *CYP1B1* mRNA levels are maintained to a large extent by “constitutively active” AHR in human breast cancer cell lines [46,76]. (2) There is a strong correlation between *AHR* and *CYP1B1* mRNA levels in the 60 breast cancer cell lines in the CCLE (*r* = 0.64; FDR < 4 × 10^−7^) and in 995 primary human breast cancers in The Cancer Genome Atlas (TCGA) (*r* = 0.27; FDR < 2.3 × 10^−16^) databases [17]. (3) While significant CYP1B1 protein was detectable by Western blotting in wild-type Hs578T, SUM149, and MDA-MB-231 cells, little or no CYP1B1 protein was detected following AHR deletion with CRISPR-Cas9 gene editing (Figure S1). GSEA analysis using the *CCLE* database demonstrated that *AHR* expression in 60 breast cancer lines is significantly and positively correlated (false discovery rate = 0.022) with the 10 most altered genes (*p* = 0.02–0.055) after AHR knockdown (Figure 3A). The one gene that was anti-correlated with *AHR* was *CDH1* (E-cadherin), the only gene in the invasion set to increase after AHR knockdown (Table 1). Similarly, there was a significant positive correlation between *CYP1B1* and the putative AHR target gene set (FDR = 0.026) and again, the one outlier with a negative correlation score was *CDH1* (Figure 3B).

To generalize results to primary human cancers, GSEA analyses was performed with the set of 10 most significantly altered genes after AHR knockdown and transcriptomic data from 977 primary human breast cancers catalogued in the TCGA database [77]. As shown for cell lines in the *CCLE*, there was a significant association (FDR = 0.018) between *AHR* expression and the set of putative AHR-regulated genes, with *CDH1* being the sole anti-correlated gene (Figure 3C). A similar outcome was observed when correlating *CYP1B1* expression with that of the AHR-regulated migration/invasion-associated genes (Figure 3D).

Thus, although multiple factors contribute to expression of the invasion-associated genes analyzed here, our data are consistent with a role for the AHR in influencing their expression in a significant fraction of primary human breast cancers. The data also support the hypothesis that AHR downregulation would be beneficial in human breast cancer.

### 2.3. AHR Inhibition Blocks Human Cancer Cell Metastasis In Vivo

**Figure 4:**
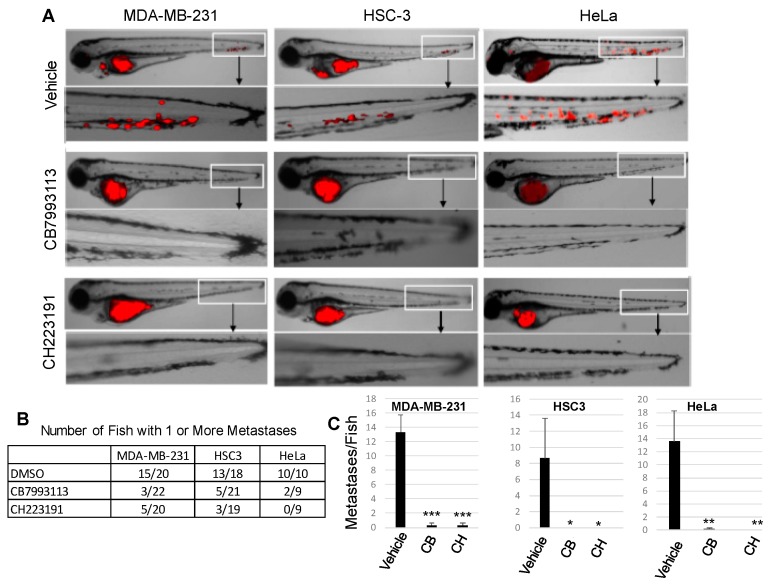
AHR inhibitors block human cancer cell metastasis in zebrafish xenograft models. Human RFP-MDA-MB-231 (TNBC), RFP-HSC3 (oral squamous cell carcinoma), or RFP-HeLa (cervical carcinoma) cells were injected into the perivitiline space of two-day-old zebrafish larvae and larvae were treated with vehicle (0.1% DMSO), 10 μM CB7993113 or 10 μM CH223191. Fish were imaged 24 h later at 8.5× magnification by confocal microscopy. (**A**) Representative images from 9–20 fish/group. (**B**) The number of fish with one or more metastases. (**C**) Data are presented as the average number of metastases/fish + SE after treatment with vehicle, CB7993113 (CB) or CH223191 (CH). * *p* = 0.08, ** *p* < 0.02, *** *p* < 0.003.

From the in vitro studies described above, it would be predicted that AHR inhibition with pharmacological agents would compromise the ability of cancer cells to metastasize in vivo. A well-characterized zebrafish model of human cancer cell metastasis [78,79,80,81,82,83] was used to test this prediction. Five hundred human RFP-MDA-MB-231 cells (TNBC), RFP-HSC3 (oral squamous cell carcinoma), or RFP-HeLa (cervical carcinoma) cells were injected into the perivitiline space of two-day-old zebrafish larvae and fish were treated with one of two AHR inhibitors in the water, 10 μM CB7993113 or 10 μM CH223191. All three human tumor cell types have significant baseline levels of AHR activity that are suppressed with AHR inhibitors [84,85,86]. Fish were imaged 24 h later by confocal microscopy. Following treatment with vehicle (0.1% DMSO), metastases in the vascular plexus of the tail (see magnification of the tail area) could readily be detected in 15 out of 20 MDA-MD-231-injected fish, 13 out of 18 HSC3-injected fish, and 10 out of 10 HeLa-injected fish (Figure 4A,B). Fish averaged between eight and 14 metastases for the three tumor types in these controls (Figure 4C). In contrast, treatment with either inhibitor dramatically reduced the number of fish with metastases, with the most profound effect being observed in larvae injected with HeLa cells treated with CH223191 (0 out of 10 fish with metastases) (Figure 4B). Accordingly, there was a significant decrease in the number of metastases per fish with inhibitor treatment for all three tumor types (Figure 4C). No overt toxicities (aberrant notochord, heart, brain morphology, larva size) were observed in these developing embryos. These in vivo results are consistent with the hypothesis that the AHR plays an important pro-tumorigenic role.

### 2.4. The Effects of AHR Agonists on Tumor Cell Invasion, Migration and Metastasis

The results described above suggest an opportunity for blocking the most lethal stages of tumorigenesis, tumor invasion, migration or metastasis, with AHR inhibitors. However, the AHR also has been reported to exhibit anti-tumorigenic effects in prostate [30], stomach [31] liver [9] pancreatic [32] and breast [33,87,88,89,90] cancers, suggesting that full or partial AHR agonists might be the more appropriate cancer therapeutic. To address this conundrum, the effects of two AHR agonists, 3,3′-diindolylmethane (DIM) and 2,3,7,8-tetrachloridibenzo-*p*-dioxin (TCDD), on cancer cell colony morphology (Matrigel), migration (scratch assay), and/or metastasis (zebrafish assay) were determined as for AHR antagonists. Both of these AHR agonists were previously reported to decrease human tumor cell growth [39,89,91,92,93].

**Figure 5:**
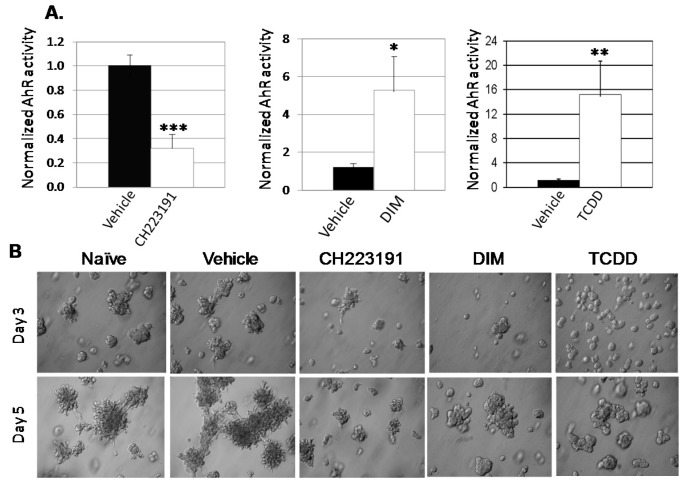
AHR inhibitor CH223191 or agonists DIM or TCDD inhibit irregular colony growth in 3D Matrigel cultures. (**A**) BP1 cells were transfected with *pGudLuc* and *CMV*-green vectors. One day later cells were treated with vehicle (DMSO), 10 μM CH223191, 5 μM DIM, or 1 nM TCDD. Normalized pGudLuc luciferase (AHR) activity was assayed 24 h later. Data pooled from 11 experiments are presented as normalized means + SE, * *p* < 0.05, ** *p* < 0.01, *** *p* < 0.001 as compared with the vehicle (0.01% DMSO)-treated controls. (**B**) BP1 cells were left untreated or treated with vehicle, 10 µM CH223191, 5 µM DIM, or 1 nM TCDD for 24 h. Cells were harvested, counted and plated in Matrigel. Representative images taken at 400× magnification on days 3 and 5 from three independent experiments, with duplicates in each experiment, are shown.

**Figure 6:**
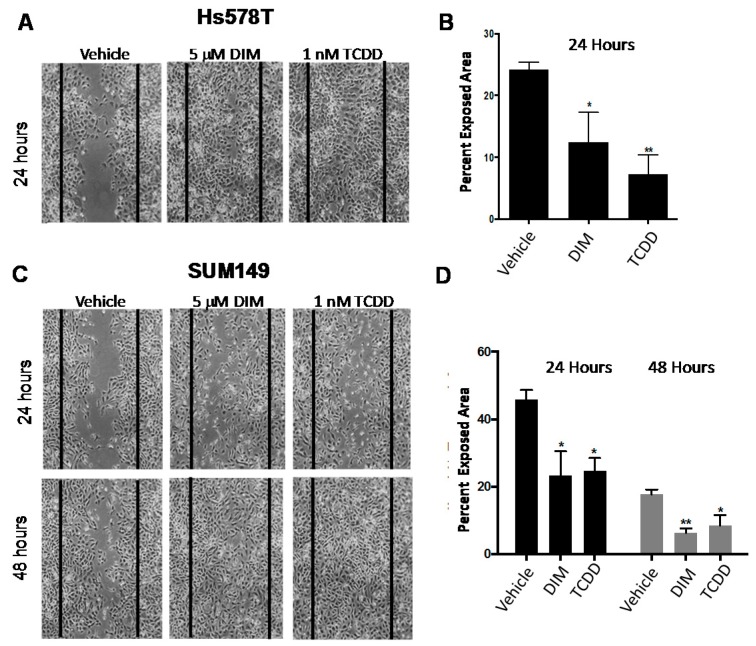
AHR agonists DIM and TCDD accelerate Hs578T and SUM149 migration. Hs578T (**A, B**) or SUM149 (**C,D**) cells were grown to confluence and scratched with a pipette tip. Cultures were then treated with vehicle (0.1% DMSO), 5 μM DIM, or 1 nM TCDD and the percent exposed area digitally quantified at 24 and 48 h. Left: Representative images at 100× magnification from a minimum of three experiments at 24 and 48 h. Vertical lines indicate the border of the scratch at time 0. Right: Data are quantified as the average percent exposed area + SE from a minimum of three independent experiments. * *p* < 0.05, ** *p* < 0.01 as compared with vehicle controls.

**Figure 7:**
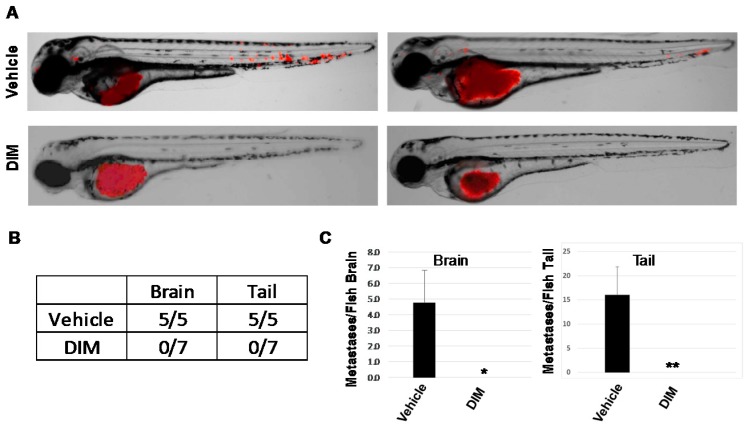
DIM inhibits metastasis in zebrafish larvae xenografts. RFP-HeLa cells were injected into the perivitiline space of two-day-old zebrafish larvae and larvae treated with vehicle (0.1% DMSO) or 1, 5, or 10 μM DIM (2–3 larvae/dose). Fish were imaged 24 h later at 8.5× magnification by confocal microscopy. (**A**) Left and right: two representative images from each group with 5–7 fish/group. (**B**) Number of fish with brain or tail metastases. (**C**) The number of metastases per fish were quantified and presented here as the average number of brain (left) or tail (right) metastases/fish + SE after treatment with vehicle (0.1% DMSO) or 1–10 μM DIM. * *p* < 0.05. ** *p* < 0.01.

As expected, 10 μM CH223191 inhibited while 5 μM DIM or 1 nM TCDD significantly enhanced AHR-dependent (pGudLuc) reporter activity in ER^−^/PR^−^/Her2^−^ BP1 cells (Figure 5A). As anticipated from earlier experiments with *AHRR*-transfected or *AHR*-specific *siRN*A-transfected BP1 cells (Figure 1), CH223191 inhibited the formation of irregular colonies characteristic of invasive cells in Matrigel (Figure 5B). Interestingly, both DIM and TCDD treatment produced a similar phenotype with a significant decrease in irregular colonies. Equal numbers of live (trypan blue-excluding) cells were recovered from these cultures indicating that none of the compounds significantly altered cell growth or viability under these 3D conditions. Interestingly, whereas AHR inhibitors significantly slowed tumor cell migration (Figure 2), both 5 μM DIM and 1 nM TCDD accelerated cell migration (reduced exposed area) of Hs578T (Figure 6A,B) or SUM149 (Figure 6C,D) cells in the scratch wound assay. These results are consistent with our previous data generated with other AHR agonists and showing that agonists accelerate this measure of tumor cell migration [17]. Finally, to compare the ability of AHR inhibitors and agonists to affect metastasis in the zebrafish assay, HeLa cells were injected into two-day-old fish larvae and the water treated with vehicle or 1, 5, or 10 μM DIM (2–3 fish at each dose). HeLa cells were chosen for these experiments since they were the most consistent of the tumors assayed, generating significant numbers of brain as well as tail metastases in all injected fish. As seen in previous studies (Figure 4), five out of five fish treated with vehicle generated significant numbers of brain and tail metastases (Figure 7A, top and Figure 7B). Reminiscent of studies with AHR inhibitors (Figure 4), DIM completely blocked metastases to the brain and tail (0 out of 7 fish with either brain or tail metastases). No changes in larvae morphology were seen. Thus, under some, but perhaps not all circumstances, AHR inhibitors and agonists can yield similar phenotypic outcomes, e.g., a decrease in irregular colony growth and metastasis.

## 3. Discussion

After several decades of scrutiny as a xenobiotic sensor, the AHR is now being evaluated as a novel target for cancer and immuno-therapy [6,15,34,35,36,37,38,39,94,95,96,97,98,99]. However, apparently conflicting results and the unpredictability of what outcomes can be expected after AHR modulation in different tissues and with different ligands (reviewed in [10,100]) has raised concerns about whether the AHR should be inhibited or hyper-activated in any given disease context. This has been particularly germane to the potential treatment of breast cancer with AHR “modulators” since both agonists and antagonists have reduced, in different systems, tumor growth. These results are reminiscent of the “Janus-faced” role of the AHR in skin disorders where both blocking and activating the AHR ameliorates disease [27]. Resolving this apparent contradiction is of considerable importance given the drive to use AHR “modulators” to treat various cancers [6,34,35,36,37,38,39,40]. Therefore, we used multiple complementary molecular and pharmacological approaches to confirm that inhibition of AHR activity suppresses aggressive tumor behavior, as defined here by a reduction in irregular colony growth in 3D Matrigel cultures, migration rate across a monolayer scratch wound, and metastasis in a zebrafish xenograft model, and to determine the effects of exogenous AHR agonists in a head-to-head comparison with the same readouts. 

The results with three TNBC lines (BP1, Hs578T, MDA-MB-231) and an aggressive triple negative inflammatory breast cancer line (SUM149), using molecular (*siAHR*, inducible *shAHR*, ectopic *AHRR* expression, and CRISPR-Cas9 gene editing) and pharmacological (AHR competitive inhibitors CH223191 and CB7993113) were completely consistent, i.e., AHR inhibition reduced all markers of tumor aggression. Furthermore, analysis of an array of invasion-associated genes after AHR knockdown suggested plausible mechanisms through which the AHR drives tumor invasion, further supporting the conclusion that AHR inhibition, at least in these TNBC and IBC lines, reduces tumor aggression. For example, AHR knockdown inversely affected *CDH1* (E-cadherin) and *Fn* (fibronectin) with *CDH1* increasing 5-fold and *Fn* decreasing 3.6-fold after AHR knockdown (Table 1). This change in gene expression is consistent with a mesenchymal to epithelial cell reversion to a less aggressive phenotype [43,71,101,102]. Indeed, suppression of *CDH1* expression forces epithelial to mesenchymal transition and imparts a cancer stem cell-like phenotype to human breast cancer cells [103]. Also of note was the significant decrease in *VCAM1* (2.6-fold), *TBS2* (5.2-fold), *MMP1* (7.7-fold), *COL14A1* (2-fold), and *MMP13* (3.3-fold), all of which have been associated with breast cancer cell invasion and metastasis to bone, lung, and/or the lymphatics [59,104,105,106,107,108,109,110,111,112]. Importantly, MMP1 and VCAM1 expression in part constitute a profile of invasive breast cancer cells [104]. AHR-dependent upregulation of MMP1 may be a characteristic that breast cancer cells share with melanoma and gastric tumor cells and with TCDD-treated normal human keratinocytes [61,62]. The mechanisms through which the AHR regulates these genes are still under study. However, the presence of five consensus AHREs within 3000 base pairs of the *CDH1* start site (−580, −700, −1100, −1230, and −1590) and two AHREs upstream of *THBS2* (−240, −280) and *MMP1* (−1380, −1478) start sites may provide an opportunity for direct AHR-mediated control of gene transcription. A second plausible mechanism is indirect regulation of invasion genes through TGF-β, previously shown to be regulated by the AHR [6,113,114] and to regulate invasion-associated genes including *MMP2* and *MMP9* [115]. Several other factors, including epiregulin [116], VAV3 [117,118], and AP1 [61] are AHR responsive and also may play a role in altering cell adhesion, motility and metastasis [104,118,119,120]. These possibilities are currently being tested.

Studies in the zebrafish model are consistent with a role for the AHR in metastasis in vivo. While the zebrafish model has significant advantages over other models of cell migration (e.g., zebrafish contain a full complement of extracellular matrix proteins that interact with and influence migratory cells and are easy to image, and the model is less expensive and more rapid than other animal models [78,82,121,122]) some caution should be exercised when extrapolating zebrafish results to human systems. The evolutionary distance between zebrafish and humans creates some uncertainty in interpretation of the results. For example, zebrafish do not express a human MMP1 homologue but do express two copies of a human MMP13 homologue [123]. Even though the AHR appears to drive expression of these two MMPs, it would be premature to conclude that AHR regulation of MMPs in specific is responsible for the failure of multiple human cancer cell lines to metastasize in zebrafish. Nevertheless, the zebrafish do generally support the notion that AHR controls cell migration in a 3D environment that more closely represents the human microenvironment than 3D Matrigel cultures. 

That these results are generalizable to other cell lines and to primary human cancers was suggested by the GSEA analyses showing a positive correlation between *AHR* or *CYP1B1* and *FN*, *VCAM1*, *THBS2*, *COL14A1*, *COL15A1*, *MMP1*, and *MMP13* and a negative correlation with *CDH1* (Figure 4). Again, although these GSEA analyses do not prove causality, they are consistent with a role for the AHR in regulation of invasion-associated genes and suggest that AHR inhibition would be a useful strategy for breast cancer therapy as suggested for other cancer types [13].

That said, both an AHR inhibitor (CH223191) and AHR agonists (DIM, TCDD) inhibited irregular colony growth (Figure 5), suggesting that both AHR agonists and antagonists could be regarded as potential therapeutics. The ability of CH223191, DIM and TCDD to moderate this invasion-associated phenotype in a single TNBC line under identical conditions argues that similar paradoxical results reported in the literature may not always reflect differences in tumor cell lines or assays, although signaling differences between the lines used in our studies and those used by others [88] have been documented [124]. Rather, we propose that both AHR inhibitors and agonists may result in a similar outcome if the inhibitors block signaling pathways driven by endogenous ligands while exogenous ligands drive different signaling pathways, effectively “diverting” the invasion signaling pathway [12]. This interpretation is consistent with the oft-cited differences in AHR signaling induced by different ligands (reviewed in [10,100]). If this hypothesis were correct, one would expect that the gene set induced by agonists such as TCDD would not represent a mirror image of those inhibited by AHR inhibitors or, put another way, endogenous ligands that drive “constitutive” AHR activity would not drive the same gene sets as exogenous ligands like DIM or TCDD. Indeed, when we used whole transcriptome microarrays to compare the genes upregulated with TCDD in SUM149 cells with those downregulated after AHR deletion by CRISPR-Cas9 editing, we saw very little overlap in genes that change significantly (FDR < 0.05), with the exception of *CYP1A1* and *CYP1B1*, genes that are consistently upregulated with both endogenous ligands (e.g., kynurenine, xanthurenic acid, FICZ) [13,17,18,125,126,127,128] and exogenous (e.g., TCDD, DIM) ligands. Results in the zebrafish metastasis assay showing that both AHR inhibitors (CH223191, CB7993113) and agonists (DIM) profoundly block TNBC (MDA-MB-231), oral squamous cell carcinoma (HSC3), and/or cervical cancer (HeLa) metastasis imply that a similar situation may exist in other cancer types. 

Even so and in keeping with sometimes unpredictable AHR-driven outcomes [10], the AHR agonists accelerated TNBC migration in the scratch wound assay (Figure 6). This result emphasizes the differences between invasion (Matrigel) and migration (scratch wound) assays where the latter requires only increased cell mobility while the former requires extracellular matrix proteolysis and degradation. While these results give one pause when considering the use of AHR agonists in breast cancer [36], they may not preclude the use of agonists since metastasis, the most lethal step in carcinomas, requires both tissue invasion and migration. 

In general, these studies add further incentive for the use of AHR inhibitors in breast and, potentially, other cancers especially given the high level of AHR expression and chronic activity in a wide variety of cancers [18,100]. Such a novel targeted approach is particularly relevant to patients with highly aggressive cancers for which few or no targeted therapeutics are available, e.g., ER^−^/PR^−^/HER2^−^ and inflammatory breast cancers. Furthermore, studies presented here directly address the agonists vs. antagonist question in the cancer setting and suggest that, depending on the context, either antagonists or agonists may serve an important unmet clinical need.

## 4. Materials and Methods

Cell culture. Hs578T, an ER^−^/PR^−^/HER2^−^ human mammary carcinosarcoma cell line [129,130] and SUM149, an inflammatory ER^−^/PR^−^/HER2^−^ breast carcinoma cell line were obtained from the ATCC (ATCC, Manassus, VA, USA). HeLa cells were authenticated by short tandem repeat DNA fingerprinting by Genetica DNA Laboratories (Burlington, NC, USA). Hs578T, SUM149, and HeLa cells were maintained in DMEM (Mediatech, Washington, DC, USA) containing 10% FBS, 19.4 mM d-glucose, 2 mM l-glutamine (Mediatech), 1% penicillin/streptomycin (Mediatech), 10 µg/mL insulin (Sigma/Aldrich, St. Louis, MO, USA) and 5 µg/mL plasmocin (Invivogen, San Diego, CA, USA). The BP1 cell line was a generous gift from Dr. J. Russo (Fox Chase Cancer Center, Philadelphia, PA, USA) [131]. BP1 cells were derived by chemically transforming non-malignant MCF-10F cells by treatment with the carcinogenic AHR ligands benzo[*a*]pyrene [131]. BP1 cells were maintained in DMEM-F/12 medium (Mediatech, Washington, DC, USA) containing 5% equine serum (Sigma/Aldrich), 20 ng/mL human recombinant EGF (Invitrogen, Carlsbad, CA, USA) and 0.5 µg/mL hydrocortisone (Sigma/Aldrich), 2 mM l-glutamine, 1% penicillin/streptomycin, 10 µg/mL insulin and 5 µg/mL plasmocin. All cell lines were cultured at 37 °C in 5% CO_2_ in a humidified incubator and grown as adherent monolayers at a maximum of 60–80% confluency. NIH guidelines for maintaining the integrity of the lines were followed.

Transient transfection. BP1 and Hs578T cells (2–5 × 10^4^) were plated in complete medium or antibiotic-free medium respectively for 24 h. BP1 cells were transfected using Mirus-TKO transfection reagent (Mirus Bio LLC, Madison, WI, USA). Hs578T cells were transfected using lipofectamine 2000 (Invitrogen). An AHRE-driven firefly luciferase reporter construct (*pGudLuc),* a generous gift from Dr. Michael Denison (University of CA, Davis, CA, USA), was used to assess AHR transcriptional activity as described [46]. Cells were co-transfected with the *pGudLuc* reporter plasmid (0.5 µg) and either renilla luciferase control *phRL-TK* (0.1 µg) or *CMV*-green (0.5 µg) to control for transfection efficiency. Where indicated, cells also were transfected with the 0.5 µg *AHRR* [132] or *pcDNA* control plasmid and incubated for 24 to 48 h. For *AHR* siRNA experiments, cells were transfected with 20 µM of *AHR* siRNA (5′-AAG UCG GUC UCU AUG CCG CTT-3′), scrambled siRNA control (5′-GCG CGC UUU GUA GGA UUC GTT-3′) (Integrated DNA Technologies, Coralville, IA, USA) or human *lamin a/c* siRNA (Qiagen, Valencia, CA, USA) and incubated for 48 h. Cells were washed with PBS, resuspended in 75 µL RPMI, and luciferase activity was determined with a Dual Glo luciferase system (Promega, Madison, WI, USA). Luminescence and eGFP fluorescence were measured using a Synergy2 multifunction plate reader (Biotek Inc., Winooski, VT, USA).

Stable, inducible AHR knockdown using a *TRIPZ shAHR vector.* Hs578T cells were stably transduced with a TRIPZ Doxycycline-inducible *shAHR* lentivirus vector (Thermo Scientific, Lafayette, CO, USA) as we previously described [21]. To produce *shAHR* lentiviral particles, 293T cells were transfected with the *shAHR* lentiviral vector along with gag/pol, rev, tat, and vsv-g (generously provided by Dr. D. Kotton, Boston University, Boston, MA, USA). Infectious supernatant containing *shAHR* was used to transduce Hs578T cells. Hs578T cells (3 × 10^3^) were plated in a 96-well plate and incubated overnight at 37 °C at 5% CO_2_ to reach 50% confluency. The following day, Hs578T cells were infected with virus solutions containing a range of MOIs (1, 10, 20 and 50) along with addition of 1 µl of 6 mg/mL hexadimethyrine bromide. Six hours post-transduction virus-containing media was replaced with 100 µL of complete media. Cells were cultured until they reached 80% confluency and then passaged. After the first passage, cells were treated with 1.5 µg/mL Puromycin (InvivoGen) for two weeks. TurboRFP expression was monitored by fluorescence microscopy 48 h after addition of 1.5 µg/mL Doxycycline (Clonetech, Mountain View, CA, USA). To confirm AHR knockdown, Hs578T cells stably transduced with *shAHR* were cultured in the presence or absence of Doxycycline for 48 h and then co-transfected with *pGudLuc* (0.5 µg) and renilla luciferase control *phRL-TK* (0.1 µg). Cells were harvested 24 h post-transfection and the pGudLuc and renilla luciferase activity was measured. Based on results from the *pGudLuc* assays, it was determined that an MOI of 50 resulted in optimal knockdown of endogenous AHR activity. For Matrigel experiments, *shAHR* Hs578T cells were either pre-treated with Doxycycline for 6 days or left untreated while in standard 2D culture. After pre-treatment, cells were harvested, counted and then plated in Matrigel.

*Lentivirus transductions and CRISPR-Cas9 gene editing.* The Red Fluorescent Protein (RFP) plasmid was cloned into PLemiR-DEST using the LR reaction gateway ligase free system (ThermoFisher/Life Technologies, San Josè, CA, USA) from the PDONR221-RFP entry clone. Lentivirus production was conducted as previously described [133]. Following lentiviral infection, Hs578T or SUM149 cells overexpressing RFP were selected in DMEM/FBS 10% containing Puromycin (2 μg/mL). The *RFP* overexpression was verified using fluorescence stereomicroscopy. The CRISPR vector lentiCRISPR v2 (Addgene no. 52961, Cambridge, MA, USA) contains Cas9 and a guide RNA cloning site (*BsmBI*). The two target sequences (5′-CCTACGCCAGTCGCAAGCGG-3′ and 5′-CCGAGCGCGTCCTCATCGCG-3′, NM_001621), selected by CRISPR designer (http://crispr.mit.edu), are located in the first exon of the *AHR*. The construct was confirmed by DNA sequencing. Cells were infected with *AHR* lentiCRISPRv2 lentivirus according to the standard protocol [134]. Cells were selected for 10 days with 2.0 μg/mL Puromycin. *AHR* deletion was confirmed by AHR-specific Western blotting and by qPCR for AHR-regulated genes in the presence or absence of an agonist (*CYP1A1* and *CYP1B1*). AHR antibody for western blotting to confirm AHR protein deletion was purchased from Cell Signaling Technologies, Danvers, MA (13790#,1:1000 dilution) and *β*-actin antibody was from Sigma-Aldrich (A5441,1:2000).

3D Matrigel assays. Matrigel basement membrane matrix (Becton/Dickinson, Bedford, MA, USA) was diluted to a concentration of 6.3 mg/mL with cold serum-free DMEM and aliquoted and stored at −80 °C. Before use, Matrigel aliquots were thawed on ice. For the bottom layer, 200 or 400 µL of Matrigel solution was added to a 24- or 12-well tissue culture plate respectively and incubated at 37 °C for 30–45 min to allow the Matrigel to solidify. Single-cell suspension containing 2–5 × 10^4^ cells in 10 μL serum-free medium was mixed with 190 µL or 390 µL of Matrigel and added to the preset Matrigel layer. Plates were incubated at 37 °C for 30–45 min to allow the Matrigel to solidify, and complete medium then was added. Medium was replaced once every two days and, where indicated, AHR agonist or antagonist was added. Following incubation at 37 °C for five to seven days, colony growth was analyzed using a Zeiss Axiovert 200 M microscope. Images were captured using a Nikon Coolpix4300 digital camera. Images shown were taken at 200× or 400× magnification.

Harvesting cells from Matrigel. Cells grown in 3D Matrigel cultures were harvested using BD Biosciences cell recovery solution. Wells were washed with cold PBS twice followed by addition of BD cell recovery solution while on ice. Cells were gently pipetted up and down to disperse the Matrigel and transferred to 15 mL falcon tubes on ice. Tubes were inverted 5–10 times and rocked for 15 min while on ice. Cells then were centrifuged at 300× *g* for 8 min. Pellets were transferred to 1.5 mL Eppendorf tubes and centrifuged at 300× *g* for 5 min followed by lysis and homogenization by addition of Buffer RLT plus (Qiagen) according to the manufacturer’s instructions.

Proliferation assays. For experiments in which cells were to be transfected, 3 × 10^4^ cells were cultured in 12-well plates for 24 h and then transfected with *AHRR,* control *pcDNA,* scrambled control siRNA or *AHR* siRNA. Cells were harvested 24 h after transfection and 3 × 10^3^ cells added in triplicate to 96-well plates. ^3^H-thymidine (10 µCi/10 µL/well) (NEN Life Science Products, Boston, MA, USA) was added 8 h later and cells were incubated for an additional 18 h. For proliferation experiments in which AHR inhibitor was used, 3 × 10^3^ cells were added in triplicate to 96-well plates and incubated 24 h in the presence of 0.1% vehicle (DMSO) or 10 µM CH223191. ^3^H-thymidine (10 µCi/10 µL/well) was added and cells were incubated for an additional 18 h. Cells were harvested onto pre-cut individual filter mats using a PHD cell harvester (Cambridge Technology Inc., Watertown, MA, USA) and ^3^H-thymidine retained on the filter mats was detected using a mini β LKB Wallac 1211 scintillation counter (Perkin Elmer, Shelton, CT, USA). For each condition in a given experiment, CPM incorporation from each of the three triplicates was averaged to generate one data point per experiment.

Boyden Chamber migration assays. BP1 and Hs578T cells (3 × 10^4^) were cultured in 12-well plates in antibiotic-free medium for 24 h. Cells were transfected with scrambled control siRNA or *AHR* siRNA oligonucleotides and incubated for 24 h. Cells were serum starved for 18 h in serum-free DMEM-F12 (BP1) or DMEM (Hs578T) medium and then washed with sterile PBS, trypsinized, and washed in DMEM serum-free medium containing 5% BSA (bovine serum albumin). Cells were counted and resuspended in serum-free medium. Cell suspensions containing 0.5 × 10^5^ cells/250 µL serum-free media were added to the Matrigel-coated Boyden chamber inserts (8 µM pores) (Millipore, Billerica, MA, USA) as per the manufacturer’s instructions. Medium containing serum (500 µL) was added to the lower chamber and the plates were incubated at 37 °C for 48 h. The invading cells (attached to the bottom of the insert membrane) were harvested after 48 h, lysed and stained with a CyQuant GR dye as per the manufacturer’s instructions and fluorescence assayed using a Synergy2 multifunction plate reader. Fluorescence emitted by the transfected cells was normalized to fluorescence emitted by the untransfected controls.

Scratch-wound assay. Cells were grown to confluence in 12-well plates. A p200 pipet tip was used to make a vertical scratch in each well and non-adherent cells were removed. Media was added and, in experiments investigating the effects of AHR inhibitors or agonists on migration, cells treated with vehicle, 10 μM CH223191, 10 μM CB7993113, 1 nM TCDD, or 5 μM DIM. Media was changed and cells were re-dosed daily. Photographs were captured at 100×. TScratch software (Tobias Gebäck and Martin Schulz, ETH Zürich) was used to quantify the closure of the scratch over time. 

Zebrafish larva metastasis assays. Zebrafish husbandry was performed as described [135] in the zebrafish facility at the Boston University School of Medicine and in accordance with IACUC-approved protocols. Zebrafish breeders were crossed and transparent zebrafish larvae isolated. For micro-injections, the RFP-labelled cells to be transplanted were trypsinized and resuspended at a concentration of 50 × 10^6^ cells/mL in DMEM with 10% FBS. One day post fertilization (1 dpf) larvae were enzymatically dechorionated using Pronase (Roche Diagnostics, Westborough, MA, USA) and allowed to recover overnight at 28 °C, in the dark, and in sterilized egg water. The 2 dpf zebrafish larvae were anesthetized with Tricaine and subsequently immobilized before injections. Borosilicate glass capillaries (1.0 mm outside diameter × 0.78 mm) (World Precision Instruments, Sarasota, FL, USA) used for micro-injection were pulled using 500 Volts (pull = 100, velocity = 250) in a capillary machine (Sutter Instrument, Novato, CA, USA). Human tumor cell transplantations were performed by injection of ~1 nL directly into the perivitiline space of the larvae using a needle holder and a micro-injection station (World Precision Instruments) as described [136]. Xenografted zebrafish larvae were then rescued in sterile egg water and transferred to a standard 6 well dishes before AHR antagonist or agonist treatment. For treatments, the transplanted zebrafish larvae were treated in the six-well plates with DMSO alone or 1 μL/mL of 1000× compounds diluted in DMSO. Twenty four hours later zebrafish larvae were individually mounted in a low melting 1.5% agarose gel (Fisher) for side view immobilization using a thin eyelash. Metastases were quantified using an Olympus MVX10 fluorescence stereomicroscope. 

*Protein extraction and western immunoblotting.* Nuclear and cytoplasmic extracts were prepared using the nuclear extraction kit (NE-PER, Thermo Scientific, Rockford, IL, USA) according to the manufacturer’s instructions. After extraction, protein concentrations were determined using a Bio-Rad protein assay (Bio-Rad, Hercules, CA, USA). Proteins were diluted with 3× sample loading buffer (100 mM Tris-Cl, pH 6.8, 200 mM dithiothreitol, 4% SDS, 0.2% bromophenol blue, 20% glycerol), boiled and 20 μg loaded onto an 8% SDS-PAGE gel. Electrophoresis was performed in a Bio-Rad Mini Protean II gel system at 100 mV for 2.5 h. After electrophoresis, gels were transferred onto nitrocellulose membranes (Bio-Rad) for 2.5 h at 150 mA. After transfer, membranes were blocked with 5% milk in TBST for 1 h. Blots were incubated overnight with mouse anti-AHR monoclonal antibody (MA1-514, Thermo Scientific), washed and then incubated 1 h with secondary goat anti-mouse Ig antibody (Thermo Scientific), washed again and visualized by enhanced chemiluminescence using a combination of 10 mL 1.25 mM luminol (Sigma/Aldrich) in 0.1 M Tris pH 8.5, 10 μL 68 mM coumaric acid (Sigma/Aldrich) in DMSO, and 30 μL 3% hydrogen peroxide (Fisher Scientific, Pittsburg, PA, USA). Blots were re-probed using β-actin-(Sigma/Aldrich) and lamin a/c-specific antibodies (Cell signaling technology, Danvers, MA, USA) to control for loading variability.

*Quantitative real time PCR.* Cells stably transduced with doxycycline-inducible *shAHR* were grown in triplicate wells in 3D Matrigel cultures for six days in the presence or absence of 1.5 µg/mL Doxycycline. Cells from triplicate wells were harvested as above, pooled and RNA extracted using an RNAeasy mini kit (Qiagen) according to the manufacturer’s instructions. Eluted RNA was quantified using a nanodrop spectrophotometer (BD). RNA (1 µg) was reverse transcribed into cDNA using a Superarray RT2 qPCR kit (SABiosciences, Fredrick, MD, USA). Real time PCR was performed using 96-well PCR array profile plates (human extracellular matrix and adhesion molecules PCR Array—PAHS-013A). Thermal cycling was performed under the following conditions on an ABI 7000 instrument: 10 min at 95 °C followed by 40 cycles of 95 °C for 15 s and 60 °C for 60 s. C_T_ values were determined using the SDS version of Applied Biosystems 1.1 analysis software and the comparative C_T_ method (Applied Biosystems, Foster City, CA, USA). Adhesion/invasion-associated genes evaluated in this assay are listed in Table 1. The relative mRNA expression of each gene was normalized against an average of five endogenous control genes (β*-actin*, β2 *microglobulin*, *HPRT1*, *RPL12A* and *GAPDH*). The ΔΔ*C*_T_ (Δ*C*_T *shAHR* (+ Dox)_ − Δ*C*_T *shAHR* (− Dox)_) values were used to calculate fold change in each experiment.

*Statistical analyses*. For experiments involving AHR activity, Boyden chamber, immunoblotting, pGudLuc reporter, proliferation, or migration assays data from a minimum of 3 independent experiments were used for statistical analysis. For multiplex qPCR experiments, cells were cultured in triplicate wells in Matrigel and pooled at the end of the incubation period and prior to mRNA isolation. Results from three independent experiments were averaged prior to statistical analysis. Statistical analyses were performed using the Student’s *t*-test (two-tailed) or one way ANOVA in combination with Tukey–Kramer multiple comparisons test to analyze the data and determine significant differences. Graphpad software (Graphpad, La Jolla, CA, USA) was used to perform one-way ANOVA.

## Figures and Tables

**Table 1:** Hs578T cells stably transduced with doxycycline-inducible *shAHR* were grown in triplicate in 3D Matrigel cultures in the presence or absence of Dox for six days. The cells from triplicate wells were harvested, pooled and RNA isolated and evaluated for expression of 84 genes encoding genes involved in cell adhesion and invasion (see Supplemental Table S1) by qPCR. The average mRNA expression for each gene was normalized to the average mRNA expression of five housekeeping genes (*β-actin, GAPDH, HPRT1, RPL13A and B2M*). Data averaged from three independent experiments are presented as relative fold change of mRNAs in Dox-treated as opposed to untreated *shAHR*-transduced Hs578T cells. Statistically significant changes were determined using a two-tailed Student’s *t*-test.

Gene	Encoded Protein	Fold Change	*p* Value
*CDH1*	E-cadherin	5.07	0.052
*FN1*	Fibronectin 1	−3.65	0.027
*VCAM1*	Vascular Cell Adhesion Protein 1	−2.58	0.039
*THBS2*	Thrombospondin 2	−5.24	0.033
*COL14A1*	Collagen Type XIV, α1	−2.05	0.005
*COL15A1*	Collagen Type XV, α1	−6.65	0.022
*MMP1*	Matrix Metalloproteinase 1	−7.71	0.029
*MMP13*	Matrix Metalloproteinase 13	−3.28	0.052

## Supplementary Materials

Supplementary materials can be found at http://www.mdpi.com/1422-0067/19/5/1388/s1.

## Abbreviations

CCLE	Cancer cell line encyclopedia
DIM	Diindolylmethane
Dox	Doxycycline
FDR	False discovery rate
FICZ	6-formyllindolo[3,2-b]carbazole
GSEA	Gene set enrichment analysis
IBC	Inflammatory breast cancer
TCDD	2,3,7,8-tetrachlorodibenzo-*p*-dioxin
TNBC	Triple-negative breast cancer
