# Supplementary material for: Towards Resolving the Pro- and Anti-Tumor Effects of the Aryl Hydrocarbon Receptor
Source: Int J Mol Sci. 2018 May 7;19(5):1388. doi: 10.3390/ijms19051388 (PMC5983651; doi:10.3390/ijms19051388)
Supplement: Supplementary file 1 [file ijms-19-01388-s001.pdf]

Supplementary

Towards Resolving the Pro- and Anti-Tumor Effects of the Aryl Hydrocarbon Receptor

Supraja Narasimhan [1], Elizabeth Stanford Zulick [2], Olga Novikov [3], Ashley J. Parks [4], Jennifer J. Schlezinger [5], Zhongyan Wang [5], Fabrice Laroche [6], Hui Feng [6], Francesca Mulas [7], Stefano Monti [8] and David H. Sherr [5,*]

1. Department of Pathology and Laboratory Medicine, Boston University School of Medicine, 72 East Concord St., Boston, MA 02118, USA; supraja.narasimhan@gmail.com
2. Biological Sciences and Biotechnology Programs, Northeastern University, Boston, MA 02115, USA; e.zulick@northeastern.edu
3. Molecular and Translational Medicine Program, Department of Medicine, Boston University School of Medicine, 72 East Concord St., Boston, MA 02118, USA; olga@novatok.com
4. Sage Therapeutics, 215 1rst St., Cambridge, MA 02142, USA; ashley.j.parks@gmail.com
5. Department of Environmental Health, Boston University School of Public Health, 72 East Concord St., Boston, MA 02118, USA; jschlezi@bu.edu (J.J.S.); wangzhy@bu.edu (Z.W.)
6. Departments of Pharmacology and Medicine, Cancer Center, Boston University School of Medicine, 72 East Concord St., Boston, MA 02118, USA; fabrice75005@gmail.com16 (F.L.); huifeng@bu.edu (H.F.)
7. Department of Pediatrics, University of California, San Diego, CA 92093, USA; fra.mulas@gmail.com
8. Division of Computational Biomedicine, Department of Medicine, Boston University School of Medicine, 72 East Concord St., Boston, MA 02118, USA; smonti@bu.edu

* Correspondence: dsherr@bu.edu, Tel.: +1-617-358-170

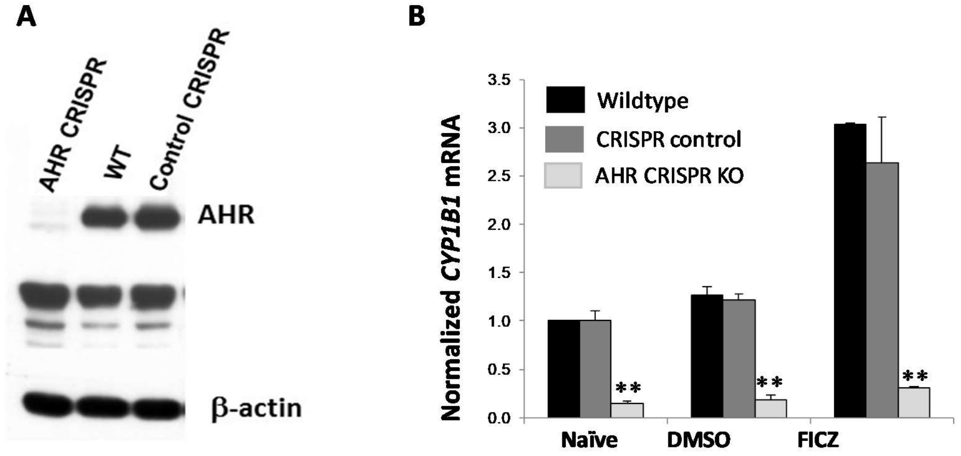

Figure S1. (**A**) A representative Western blot for AHR and α-actin expression in wild-type (WT) control CRISPR-Cas9-transduced, or *AHR*-specific CRISPR-Cas9- transduced Hs578T cells. (**B**) *CYP1B1* expression, as assayed by qPCR, in wild-type (WT), control CRISPR-Cas9-transduced, or *AHR*-specific CRISPR-Cas9-transduced Hs578T cells treated for 48 h with nothing, DMSO (0.1%) or AHR agonist (0.5 mM 6-formylindolo[3,2-barvazole (FICZ)). Data are averages from three independent experiments + SE. **<0.01.

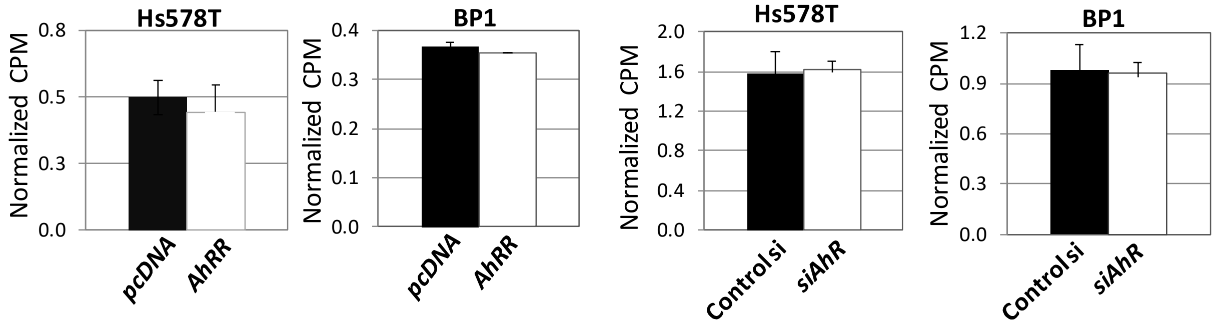

Figure S2. Hs578T and BP1 cells were transfected with control *pcDNA*, *AHRR* control scrambled *siRNA*, or *AHR siRNA* (*siAHR*), harvested after 24 h, and plated in triplicate in 96-well plates. Cells were allowed to adhere for 20 h prior to the addition of ^{3}H-thymidine. Wells were harvested individually 18 h later and the incorporated CPM measured. In each experiment, data were normalized to ^{3}H-thymidine incorporation in untransfected cells. Data pooled from 3–4 independent experiments are presented as normalized means + SE. No significant differences in CPM incorporation were seen.

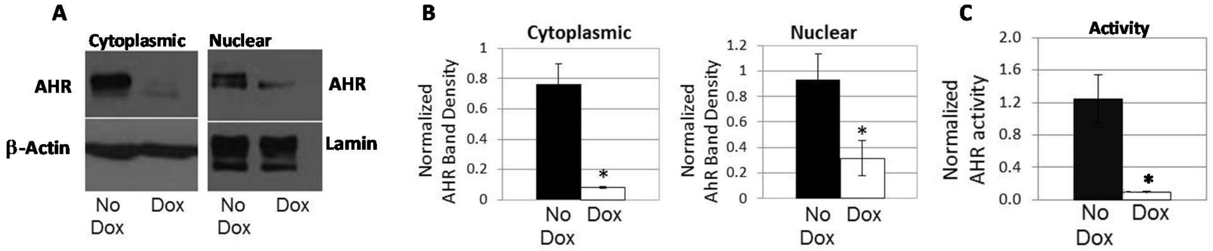

Figure S3. Doxycycline-inducible *shAHR* decreases AHR expression and activity in Hs578T cells. (**A**) Hs578T cells stably transduced with Doxycycline (Dox)-inducible *shAHR* were cultured for six days in the presence or absence of Dox (1.5 μg/ml). Cells were harvested and cytoplasmic and nuclear cell extracts (20 μg protein) probed for AHR, β-actin and lamin a/c by immunoblotting. Data are representative of three independent experiments. (**B**) AHR protein bands were normalized to β-actin or lamin a/c. Data presented are the mean band densities from three independent experiments + SE, *$p < 0.03$ as compared with the "No Dox" control group. (**C**) Dox-inducible *shAHR*-transfected cells were plated in duplicate wells and cultured for four days in the presence or absence of Dox. At the end of day 4, cells were harvested, counted, and plated in 24-well plates in the presence or absence of Dox and incubated overnight. The cells then were transfected with *pGudLuc* and *phRL-TK* and incubated for 24 h. Cells were harvested and normalized AHR-driven luciferase (AHR) activity assayed as in Figure 1. Data from six independent experiments are presented as mean normalized luciferase (AHR) activity + SE, *$p < 0.02$ using the Student's t-test as compared with the "No Dox" control group.

Functional gene grouping	Genes
Cell adhesion molecules	Transmembrane molecules: CD44, CDH1, HAS1, ICAM1, ITGA1, ITGA2, ITGA3, ITGA4, ITGA5, ITGA6, ITGA7, ITGA8, ITGAL, ITGAM, ITGAV, ITGB1, ITGB2, ITGB3, ITGB4, ITGB5, MMP14, MMP15, MMP16, NCAM1, PECAM1, SELE, SELL, SELP, SGCE, SPG7, VCAM1.
	Cell-cell adhesion: CD44, CDH1, COL11A1, COL14A1, COL6A2, CTNND1, ICAM1, ITGA8, VCAM1
	Cell-matrix adhesion: ADAMTS13, CD44, ITGA1, ITGA2, ITGA3, ITGA4, ITGA5, ITGA6, ITGA7, ITGA8, ITGAL, ITGAM, ITGAV, ITGB1, ITGB2, ITGB3, ITGB4, ITGB5, SGCE, SPP1, THBS3
	Other adhesion molecules: CNTN1, COL12A1, COL15A1, COL16A1, COL5A1, COL6A1, COL7A1, COL8A1, VCAN, CTGF, CTNNA1, CTNNB1, CTNND2, FN1, KAL1, LAMA1, LAMA2, LAMA3, LAMB1, LAMB3, LAMC1, THBS1, THBS2, CLEC3B, TNC, VTN
Extracellular matrix proteins	Basement membrane constituents: COL4A2, COL7A1, LAMA1, LAMA2, LAMA3, LAMB1, LAMB3, LAMC1, SPARC
	Collagens and ECM structural constituents: COL11A1, COL12A1, COL14A1, COL15A1, COL16A1, COL1A1, COL4A2, COL5A1, COL6A1, COL6A2, COL7A1, COL8A1, FN1, KAL1.
	ECM proteases: ADAMTS1, ADAMTS13, ADAMTS8, MMP1, MMP10, MMP11, MMP12, MMP13, MMP14, MMP15, MMP16, MMP2, MMP3, MMP7, MMP8, MMP9, SPG7, TIMP1.
	ECM protease inhibitors: COL7A1, KAL1, THBS1, TIMP1, TIMP2, TIMP3.
	Other ECM molecules: VCAN, CTGF, ECM1, HAS1, SPP1, TGFBI, THBS2, THBS3, CLEC3B, TNC, VTN.

Table S1. Genes evaluated by qPCR using the RT21 superarray qPCR kit (SABiosciences) for regulation after AHR knockdown with Dox-inducible *shAHR* RNA.
